# A Survey of Jellyfish Sting Knowledge among Naval Personnel in Northeast China

**DOI:** 10.3390/ijerph13070725

**Published:** 2016-07-19

**Authors:** Ting Kan, Li Gui, Wenwen Shi, Yan Huang, Shuang Li, Chen Qiu

**Affiliations:** Department of Emergency Nursing, School of Nursing, Second Military Medical University, Shanghai 200433, China; ting.kan@smmu.edu.cn (T.K.); xiaowenz@126.com (W.S.); yan8811@hotmail.com (Y.H.); shiny0820@foxmail.com (S.L.); qiuchenqiuying@163.com (C.Q.)

**Keywords:** naval personnel, jellyfish sting, knowledge, medical education, occupational and environmental health

## Abstract

**Background:** Jellyfish envenomation is common along the coastal area, and can cause severe consequences. Naval personnel are among the high-risk population for this injury. The aim of this study was to assess knowledge regarding jellyfish envenomation among naval personnel in a navy unit in northeast China. **Methods:** A predesigned questionnaire was distributed to 120 naval members in January 2015. The data of 108 respondents were included in the statistical analysis. **Results:** We found that 38.0% of the respondents selected jellyfish sting as the common wound in their units, and 13.0% had experienced or observed this injury. In addition, 63.0% of the participants rated their own knowledge as “low” or “none”. The average score they got was 5.77 ± 2.50, with only 16.7% getting a score above 60% of the full score. The correct rates of five questions were below 60%. No statistical differences existed in the knowledge score among different groups of respondents defined by socio-demographic variables. **Conclusions:** Jellyfish sting is common in this navy unit, but personnel got a low score on the knowledge assessment. They also lacked confidence in first aid. Medical education and training should be implemented to address this issue.

## 1. Introduction

Jellyfish are invertebrates distributed in the marine ecosystem throughout the world. Although most of them are harmless, venomous species can be dangerous to humans. Jellyfish are composed of a bell-shaped body and tentacles that are covered with tens of thousands of specialized cells, called cnidocytes. When the hair-like trigger of a cnidocyte is activated by mechanical or chemical stimulation, the organelles in the cnidocyte, called nematocysts, fire stinging barbs and inject venom into the victim [1,2]. This process takes no more than a few microseconds [3], and the nematocysts can discharge even when separated or if the jellyfish is dead [4].

Signs and symptoms caused by jellyfish envenomation vary by the species, the individuals and the amount of exposed skin. Some sting cases are mild without permanent sequelae, while some result in severe consequences including persistent pain, urticaria, vesicular formation, superficial necrosis [5], eye injuries [6], cardiovascular reactions [7], Irukandji syndrome [8], multiple organ dysfunction [9], and even death [10]. As most jellyfish sting cases go unreported, accurate data about the incidence is hard to obtain. There are an estimated 150 million jellyfish envenomation cases annually [11]. According to the Surf Life Saving Australia (SLSA) 2014–2015 annual report, there were 23,500 cases of first aid for marine envenomation emergencies in Australia in the report year [12]. A study in southern Italy estimated that approximately 400,000 Euros were spent on jellyfish-related medical services along Italian coasts during a five-year period [13]. Therefore, it has become a serious public health issue and increases the burden on medical services.

Jellyfish sting cases have been well described in tropical and subtropical areas, since such areas are more suitable for jellyfish to grow and propagate. However, a significant increase in jellyfish blooms has been observed over the last decade in temperate regions, especially in China, due to intensive human activity, such as overfishing, and contributing to eutrophication and global warming [14,15]. *Aurelia aurita*, *Nemopilema nomurai*, *Cyanea capillata* and *Cyanea nozakii* form the outbreaks in the temperate Chinese sea [15]. *Aurelia aurita* (moon jellyfish) is distributed worldwide in the coastal waters [15]. It was thought to be relatively harmless in previous studies, but the stinging case reports and the toxicological study now demonstrate it is venomous to humans [16,17]. The other three species are well-known poisonous jellyfish. *Nemopilema nomurai* is mostly distributed in the East Asian marginal seas, including the Bohai Sea, Yellow Sea, Northern East China Sea and the seas of Korea and Japan [18]. *Cyanea capillata* (Lion’s mane jellyfish) is distributed worldwide and is more common in the North Sea, North Atlantic, Arctic Sea, and North Pacific [19]. *Cyanea nozakii* blooms were observed in the Northern East China Sea, Yellow Sea and Bohai Sea [20].

The increasing number of this species, together with its invisibility in water, poses a threat to the health of saltwater recreational travelers and other people who participate in sea activities. Naval personnel are organized to train regularly in the sea, and thus are among the high-risk population who are exposed to jellyfish envenomation. We conducted a survey on the knowledge about environmental emergencies among naval personnel in a specific navy unit in northeast China. The comprehensive survey consisted of multiple sub-questionnaires. This paper reports the findings of one part regarding the jellyfish sting.

## 2. Materials and Methods

### 2.1. Design and Sample

In January 2015, one author went to a navy unit in northeast China to investigate the health status and health knowledge of naval personnel. A cross-sectional study design was adopted to assess their knowledge about jellyfish envenomation. The target population of this study was active-duty naval personnel in the unit. Participants were recruited using convenience sampling. Naval members who had health care work experience were excluded from data analysis.

### 2.2. Data Collection

A questionnaire (see Supplementary Material) was developed based on the Cochrane systematic review [21] and 2014 Expert Consensus released by the Chinese Society of Toxicology [22]. The questionnaire consisted of two sections: (a) socio-demographic information, including age, gender, nationality, military-related information, education level, and medical background; (b) knowledge about jellyfish envenomation, including knowledge sources, experience of jellyfish sting, emergency first response (EFR) for jellyfish sting, general knowledge, knowledge about symptoms and treatment of jellyfish sting, and risk factors of severe envenomation. The knowledge section comprised 10 multiple-choice questions requiring single or multiple answers, three true-false items and one open question. 

Jellyfish in this questionnaire refers to *Aurelia aurita*, *Nemopilema nomurai*, *Cyanea capillata* and *Cyanea nozakii*. Therefore, the correct answers to questions, including venomous parts, periods of high incidence and first aid treatment, were determined according to specific knowledge regarding these four species.

### 2.3. Data Analysis

The SPSS for Mac version 20.0 program (IBM Corporation, Chicago, IL, USA) was employed for data analysis. Categorical variables were described using counts and percentages. The correct answer of true-false items and multiple-choice questions requiring a single answer counted as 1 point, otherwise 0. The scoring method of multiple-choice questions requiring multiple answers was as followed: if a wrong option was ticked, it counted as 0, otherwise each right option increased by 1 point. Mean ± standard deviation was adopted to describe the knowledge score. A knowledge score above 60% of the full score was acceptable, or it was unsatisfactory. Univariate analyses of each factor that potentially affect the total knowledge score were conducted to clarify the associations between the total knowledge score and the potential variables. As the knowledge score did not conform to normal distribution, non-parametric tests were used to conduct the univariate analyses. The level of statistical significance was taken as being *p* < 0.05.

### 2.4. Ethical Consideration

All questionnaires were filled out voluntarily and anonymously. The study was conducted in accordance with the Declaration of Helsinki, and ethical approval of this study was obtained from the Institutional Review Board of the Second Military Medical University (CHEC2015-037), and the confidentiality of the data collected was strictly maintained. Each participant was asked to sign a written consent form in Chinese that outlined the purpose, procedures and duration of the study.

## 3. Results

A total of 120 naval members were approached and invited to join the study. Seven respondents did not complete the questionnaires, and five had worked in health care settings, leaving 108 (90%) in the final analysis. The socio-demographic information of the participants is shown in Table 1. All of the participants were male, and 87% were below 30 years. Most (79.6%) had served in the army less than 10 years. Nearly 87% received an education below a bachelor degree.

Thirty-eight percent of the respondents (*n* = 41) selected jellyfish sting as a common wound in their units, ranking second only to mosquito bites (84.3%, *n* = 91). Other common wounds in naval units included bee sting (33.3%, *n* = 36), dog bites (31.5%, *n* = 34), and sea snake bites (11.1%, *n* = 12). Thirteen percent (*n* = 14) reported they or their companion had experienced a jellyfish sting. When this happened, six out of the 14 (42.9%) naval members called for surgeons or medical corpsmen, four (28.6%) took simple interventions immediately, and three (21.4%) called for help and took some interventions. 

Most participants (63.0%, *n* = 68) rated as “low” or “none”, whereas only four (3.7%) rated as “expert” or “high”, and the others were “moderate”. Military medical education (25%, *n* = 27) and books/magazines/newspapers (25%, *n* = 27) were the most frequently used sources for naval personnel gaining information about jellyfish stings. Other information sources, including television, families/friends and the Internet, were chosen by 16 (14.8%), 14 (13.0%), and eight (7.4%) respondents, respectively.

Table 2 provides the details of the naval personnel’s general knowledge about jellyfish envenomation. The scores of this part ranged from 0 to 6, and the average was 2.93 ± 1.13. There were three correct answers for the “high-incidence periods” question, so the total percentage exceeded 100 and a “correct answer” row was added. Two respondents ticked more than one option for the “envenomation part” question, so the total percentage also exceeded 100. The results of the non-parametric tests showed that no statistical differences existed in the knowledge score among different groups of respondents defined by socio-demographic variables. 

Table 3 shows respondents’ answers regarding the manifestation of jellyfish envenomation. The highest score they got was 7, but the average score just reached 1.88 ± 1.80. There were multiple correct answers for these two questions, so the total percentage exceeded 100 and a “correct answer” row was added. No statistical differences existed in the knowledge score among different groups of respondents defined by socio-demographic variables.

Table 4 presents respondents’ judgment on the first aid for jellyfish envenomation. The average score was 0.96 ± 0.68, whereas those who answered both questions correctly could get 2. The knowledge score of this part did not differ significantly among respondents with different socio-demographic characteristics.

The expected total score of all the knowledge questions ranged from 0 to 15. The highest score the naval members got was 12, and the lowest was 1. The average score stood at 5.77 ± 2.50, with only 16.7% respondents (*n* = 18) getting a score ≥9 (60% of the full score). No statistical differences existed in the knowledge score among different groups of respondents defined by socio-demographic variables. 

As for the preventive measures for jellyfish stings, only 11 participants (10.2%) responded to this open-ended question. The possible effective measures they proposed included “do not train when and where jellyfish bloom” (*n* = 3), “learn self-aid knowledge about jellyfish stings” (*n* = 2), “forbid swimming without permission” (*n* = 2), “avoid swimming in the sea” (*n* = 2), “wear protective suit” (*n* = 1), “clear jellyfish before training” (*n* = 1), “do not touch jellyfish” (*n* = 1), “train in company with surgeon” (*n* = 1), and “report immediately when someone suffers sting” (*n* = 1).

## 4. Discussion

To our knowledge, this is the first study to survey jellyfish sting knowledge among a specific group. We conduct the survey in a navy unit in northeast China. Although the jellyfish sting was the second most common injury listed by the naval members, less than half of those who have experienced or observed a jellyfish sting could take simple interventions immediately. In addition, most of them were not confident in their own knowledge about jellyfish stings. According to the statistics, their actual knowledge level was suboptimal as well.

### 4.1. General Knowledge about Jellyfish Envenomation

Since the dead jellyfish or separated tentacles can still cause envenomation if they are wet [4], naval members must be cautious around them and should not touch them with bare hands for any reason. Jellyfish would not attack people actively and stings normally occur after an encounter with swimmers, so people should keep away from unknown soft-bodied creatures when swimming in the sea. It would also reduce the morbidity of jellyfish stings if seawater activities and sea training did not occur in high-incidence periods.

General knowledge of naval personnel in this study, however, was not as satisfactory as expected. The rates of the correct answers for two questions were below 60%, and that of the “high-incidence period” was the lowest. The majority of naval members were aware that jellyfish stings occur frequently in summer, but less than 30% participants knew there were also numerous jellyfish sting cases during nighttime and after the rain. Jellyfish tend to move towards the seaside after the rain, as they are attracted by fresh water [22].

Further education should focus on these knowledge points to make up for their shortage and to eliminate their misunderstanding. Jellyfish sting cases may be prevented effectively if naval personnel have a better understanding of the general knowledge regarding how the jellyfish cause envenomation and the high-incidence periods. Further studies could be designed to explore the impact of knowledge improvement on the morbidity from jellyfish stings.

### 4.2. Manifestation Recognition

Early recognition of jellyfish stings contributes to targeted first aid. A local skin reaction, such as itching and burning pain, is the symptom of mild jellyfish envenomation. However, if the victim experiences hoarseness, chest pain or pallor, it indicates that allergic shock may have developed. A few cases progress rapidly to acute pulmonary edema and allergic shock within tens of minutes to six hours, and sudden death could also occur [23]. Therefore, identification of risk factors for severe cases is necessary. Victims who are in the following situations must be sent to the hospital and observed closely: (a) allergy constitution; (b) older than 65 years; (c) history of heart disease; (d) large stung area; and (e) body temperature ≥38 °C [22].

This study suggests that most naval personnel have misunderstandings of jellyfish sting manifestation. Less than 30% of participants identified local itching and burning pain as symptoms of mild envenomation, although many ticked one or the other. Nearly 60% of participants chose hoarseness, chest distress or pallor as mild case symptoms, which would delay the resuscitation of allergic shock and might lead to death. Recognition rates of risk factors for exacerbation were below 60%, except allergic constitution. 

### 4.3. Knowledge about First Aid

Victims who are stung by jellyfish should go ashore immediately and clean the wound with seawater. Fresh water must be avoided, as the osmotic pressure is low, thus causing nematocysts to burst and release toxins [24]. The next step is to remove the tentacles and nematocysts that remain in the skin. It has been recommended to cover the wound with a paste of seawater, or even dry sand, and then remove the tentacles using a knife, forceps, tweezers, or manually while wearing rubber gloves or covering the wound with towels or clothes [25]. However, bare hands are forbidden, in case hands suffer secondary envenomation. Naval personnel did not perform well on this part either. Approximately 65% of participants mistook fresh water as the irrigation fluid to clean sting wounds. The use of fresh water would undoubtedly exacerbate the wound and cause heavier injuries. A similar proportion of participants knew they could not pull out nematocysts without protection of the hands.

Besides, there are some measures that could or could not be taken after the first aid interventions. These should be also emphasized in future health education. Hot water immersion was proved beneficial to *Chironex fleckeri* envenomation [26], and could relieve pain caused by *Carybdea alata* [27,28], *Carukia barnesi* [28], and *Physalia physalis* (the Portuguese Man-of-War) [29,30]. However, another study showed that it was ineffective for *Physalia physalis* and *Chrysaora quinquecirrha* (the “sea nettle”) [31]. A folk remedy using vinegar has sometimes been tried. It did work for victims stung by *Chironex fleckeri* [32], *Carukia barnesi* [25], *Carybdea rastoni* [33], and *Morbakka* [34]. However, evidence also demonstrated that vinegar would increase the venom release of *Carybdea alata*, *Chrysaora quinquecirrha* [32], *Pelagia noctiluca* [35] and *Cyanea capillata* [36]. The effect of vinegar on *Chiropsalmus quadrumanus* [4,32] and *Physalia physalis* [4,31,37] remained controversial. Pressure immobilization bandaging (PIB) for jellyfish envenomation remains controversial and is considered potentially dangerous. A considerable amount of venom might remain in not-yet-discharged nematocysts adhered to the victim’s skin, and PIB may stimulate additional venom discharge from them [25].

### 4.4. Preventive Measures

There are many measures that can prevent jellyfish envenomation. Personal protective equipment (PPE) is almost totally effective against all jellyfish stings and is routinely recommended for all people who participate in activities in risky sea areas [38]. A stinger net could also be located to keep jellyfish out of frequently used sea areas. This is effective for large-size jellyfish including the four main species distributed in Chinese coastal areas, but for small-size jellyfish, such as Irukandji, the stinger net would not work. Adequate signage should be placed at sea training beaches to notify naval personnel about the jellyfish risk. If economic conditions allow, jellyfish sting inhibitor cream could be used as a prophylactic treatment, which reduces the risk of people developing symptoms after exposure to jellyfish tentacles [11,39].

However, the response rate of the open-ended question regarding preventive measures was very low. Most measures proposed were useful, such as avoid training or swimming during high-incidence periods, wear a protective suit, and acquire more knowledge. However, some were extreme, for example avoiding swimming in the sea.

### 4.5. Medical Education and Training

Naval personnel listed military medical education as the most frequently used information source for jellyfish envenomation. It implies that well-designed medical education and training are needed to convey the correct knowledge systematically and comprehensively. Some members also searched for information from books/newspapers/magazines, so brochures may serve as effective education and training methods. Other methods are also needed to improve their knowledge about manifestation and first aid treatment. Photographs of skin reactions could serve as an effective method for manifestation education, and skill training is necessary for first aid education. Case studies could be selected as a training method for comprehensive exercises.

Judging from the low level of jellyfish knowledge among naval personnel in this study, it is reasonable to infer that other exposed groups may also lack relevant knowledge. Further studies among other population, such as fishermen and seaside travelers, could be conducted to verify this inference. Taking this into consideration, medical education regarding jellyfish and their stings aimed at the general population may be also needed.

## 5. Limitations

There are several limitations of our study. Firstly, selection bias resulting from the small sampling size and convenience sampling devalued the representativeness of the sample. Secondly, as participants were recruited from only one military unit, generalization of the study findings is restricted. It could be inferred that the general population had a similar level of jellyfish knowledge, but further studies are needed to verify this.

## 6. Conclusions

As far as we know, this is the first study to survey knowledge about jellyfish envenomation among a specific high-risk population. The jellyfish sting is common in this naval unit in northeast China, but personnel got low scores on knowledge assessment regarding symptoms, risk factors and first aid treatment of jellyfish stings. It implies that comprehensive medical education and training should be constructed to address this issue. In addition, feasible preventive measures could be taken to protect naval personnel from jellyfish envenomation.

## Figures and Tables

**Table 1:** Socio-demographic characteristics of respondents (*n* = 108).

Characteristics	*n* (%)
Gender	
Male	108 (100.0)
Female	0 (0.0)
Age (years)	
≤20	21 (19.4)
21–30	73 (67.6)
31–39	14 (13.0)
Military Service Time (years)	
1–5	55 (50.9)
6–10	31 (28.7)
11–15	15 (13.9)
16–20	6 (5.6)
No response	1 (0.9)
Education level	
Bachelor degree or above	14 (12.9)
College degree	42 (38.9)
Vocational degree	23 (21.3)
Senior high school diploma	26 (24.1)
Junior high school diploma or below	1 (0.9)
No response	2 (1.9)
Relatives or friends work in healthcare field	
Yes	43 (39.8)
No	63 (58.3)
No response	2 (1.9)

**Table 2:** General knowledge about jellyfish envenomation (*n* = 108).

Items	*n* (%)
Which part of jellyfish would cause envenomation?	
① Body	13 (12.0)
② Tentacles *	81 (75.0)
③ Oral arms	7 (6.5)
No response	9 (8.3)
The correct answer: ②	80 (74.1)
Jellyfish could cause envenomation even if they are separated or dead.	
True *	65 (60.2)
False	42 (38.9)
No response	1 (0.9)
Jellyfish would not attack people actively unless they are provoked.	
True *	53 (49.1)
False	55 (50.9)
Which of the following are high-incidence periods of jellyfish sting?	
① Noontime	15 (13.9)
② Nighttime *	18 (16.7)
③ After the rain *	31 (28.7)
④ Summer *	87 (80.6)
⑤ Spring	7 (6.5)
No response	3 (2.8)
The correct answer: ②③④	4 (3.7)

* The correct answer.

**Table 3:** Knowledge about the manifestation of envenomation (*n* = 108).

Items	*n* (%)
What are the symptoms of mild envenomation?	
① Local itching *	74 (68.5)
② Burning pain *	69 (63.9)
③ Hoarseness	8 (7.4)
④ Chest distress	40 (37.0)
⑤ Pallor	16 (14.8)
No response	3 (2.8)
The correct answer: ①②	32 (29.6)
Which of the following situations indicates the patient should be sent to hospital immediately to prevent exacerbation?	
① Obesity	11 (10.2)
② Allergic constitution *	73 (67.6)
③ Having a cold	36 (33.3)
④ Older than 65 years *	31 (28.7)
⑤ Heart diseases history *	35 (32.4)
⑥ Large stung area *	63 (58.3)
⑦ Body temperature ≥ 38 °C *	40 (37.0)
⑧ Local itching	22 (20.4)
No response	3 (2.8)
The correct answer: ②④⑤⑥⑦	4 (3.7)

* The correct answer.

**Table 4:** Knowledge about first aid (*n* = 108).

Items	*n* (%)
What would you do with the wound if someone suffered a jellyfish sting?	
Go ashore and clean with sea water *	34 (31.5)
Go ashore and clean with fresh water	71 (65.7)
No response	3 (2.8)
The nematocyst that remained in skin should be pulled out with bare hands.	
True	37 (34.3)
False *	70 (64.8)
No response	1 (0.9)

* The correct answer.

## Supplementary Materials

The following are available online at www.mdpi.com/1660-4601/13/7/725/s1, Table S1: Socio-demographic information, Table S2: Knowledge about jellyfish envenomation.
