# Supplementary material for: A Survey of Jellyfish Sting Knowledge among Naval Personnel in Northeast China
Source: Int J Environ Res Public Health. 2016 Jul 19;13(7):725. doi: 10.3390/ijerph13070725 (PMC4962266; doi:10.3390/ijerph13070725)
Supplement: Supplementary file 1 [file ijerph-13-00725-s001.pdf]

Supplementary Materials: Survey of Jellyfish Sting Knowledge among Naval Personnel in Northeast China

Ting Kan, Li Gui, Wenwen Shi, Yan Huang, Shuang Li and Chen Qiu

Jellyfish Envenomation Knowledge Questionnaire

We are conducting a survey regarding naval personnel's knowledge about jellyfish envenomation. We ask if you could please spend 10 min to help fill out this questionnaire. The information you provide is completely confidential and will be only used for academic research. Therefore, your participation and the truthful answers based on your own condition are important for us. Thank you for the support.

Table S1. Socio-demographic information.

No.	Questions-Stems	Options/Answers
A.1.	What is your gender?	□ Male □ Female
A.2.	How old are you?	____________
A.3.	What is your nationality?	____________
A.4.	How many years on duty?	____________
A.5.	What is your unit?	____________
A.6.	What is your military rank?	____________
A.7.	What is your post of duty?	____________
A.8.	What is the highest education level that you have obtained?	□ Bachelor's degree or above □ College degree □ Vocational degree □ High school diploma □ Junior high school diploma or below
A.9.	Have you ever worked in medical or healthcare filed?	□ Yes □ No
A.10.	Is there any medical staff in your family, relatives or friends?	□ Yes □ No

Table S2. Knowledge about jellyfish envenomation.

No.	Questions-Stems	Options/Answers
B.1.	How would you rate your knowledge about treatment measures for jellyfish sting?	□ 5-expert □ 4-High □ 3-Moderate<br>□ 2-Low □ 1-None (If you choose "none", please skip to B.3.)
B.2.	Where did you obtain the knowledge about jellyfish sting?	□ Military medical education □ Television □ Books/Magazines/Newspapers □ Internet □ Families/Friends □ Others: ________
B.3.	Have you or your companions ever experienced jellyfish sting?	□ Yes (Number of times: _____)<br>□ No (If you choose "no", please skip to B.5.)
B.4.	What was your first reaction when you or your companions suffered jellyfish sting?	□ Too nervous to do anything<br>□ Call for surgeon or medical corpsmen<br>□ Take simple interventions immediately
B.5.	Which part of jellyfish would cause envenomation?	□ Body □ Tentacles □ Oral arms
B.6.	What are the symptoms of mild envenomation? (You can tick more than one option)	□ Local Itching □ Burning pain<br>□ Hoarseness □ Chest distress □ Pallor
B.7.	What would you do with the wound if someone suffered jellyfish sting?	□ Go ashore and clean with sea water<br>□ Go ashore and clean with fresh water
B.8.	After jellyfish sting, which of the following situations indicates the patient should be sent to hospital immediately to prevent exacerbation? (You can tick more than one option)	□ Older than 65 years □ Obesity<br>□ Allergic constitution □ Having a cold<br>□ Cardiac diseases history □ Large stung area<br>□ Body temperature ≥ 38℃ □ Local Itching
B.9.1.	Jellyfish could cause envenomation even if they are separated or dead.	□ True □ False
B.9.2.	Jellyfish would not attack people actively unless they are provoked.	□ True □ False

B.9.3	The nematocyst that remained in skin should be pulled out with bare hands.	□ True □ False
B.10.	Which of the following are high-incidence periods of jellyfish sting? (You can tick more than one option)	□ Noontime □ Nighttime □ After the rain<br>□ July and August in summer<br>□ March and April in spring
B.11.	Please list measures that could prevent jellyfish envenomation as many as possible. (Such as setting up warning signs in sea training sites)	____<br>____<br>_________________
B.12.	Which of the following bites or envenomation are common in your unit?	□ Sea snakes □ Spiders □ Mosquitoes<br>□ Scorpions □ Bees □ Centipedes<br>□ Jellyfish □ Dogs □ Others: ________

Thank you very much for your help in completing this questionnaire!

© 2016 by the authors; licensee MDPI, Basel, Switzerland. This article is an open access article distributed under the terms and conditions of the Creative Commons by Attribution (CC-BY) license (http://creativecommons.org/licenses/by/4.0/).
